# The association of parent’s outcome expectations for child TV viewing with parenting practices and child TV viewing: an examination using path analysis

**DOI:** 10.1186/s12966-015-0232-2

**Published:** 2015-05-28

**Authors:** Lauren Johnson, Tzu-An Chen, Sheryl O Hughes, Teresia M O’Connor

**Affiliations:** Menninger Department of Psychiatry and Behavioral Sciences, Baylor College of Medicine, Houston, Texas USA; Children’s Hospital, New Orleans, LA USA; USDA/ARS Children’s Nutrition Research Center, Department of Pediatrics, Baylor College of Medicine, Houston, Texas USA; Academic General Pediatrics, Department of Pediatrics, Baylor College of Medicine, Houston, Texas USA

**Keywords:** Television, Screen media, Parenting, Outcome expectations, Child

## Abstract

**Background:**

Television (TV) viewing has been associated with many undesirable outcomes for children, such as increased risk of obesity, but TV viewing can also have benefits. Although restrictive parenting practices are effective in reducing children’s TV viewing, not all parents use them and it is currently unclear why. The current study examined parenting practices related to TV viewing in the context of social- cognitive theory. Specifically, we hypothesized that positive and negative Parental Outcome Expectations for child’s TV Viewing (POETV) would be associated with social co-viewing and restrictive parenting practices, and that POETV and parenting practices influence the amount of TV viewed by child.

**Method:**

Data were collected from an internet survey of 287 multi-ethnic parents and their 6–12 year old children on participants’ sociodemographic information, parenting practices related to TV use, POETV, and parent and child TV viewing. Path analysis was used to examine the relationship amongst variables in separate models for weekday and weekend TV viewing. controlling for child age, household education, and parental TV viewing.

**Results:**

The results provided partial support for the hypotheses, with notable differences between weekday and weekend viewing. The models explained 13.6 % and 23.4 % of the variance in children’s TV viewing on weekdays and weekends respectively. Neither positive nor negative POETV were associated with restrictive TV parenting in either model. One subscale each from positive and negative POETV were associated with social co-viewing parenting on both weekends and weekdays in the expected direction. Restrictive parenting practices were directly negatively associated with children’s TV viewing on weekdays, but not weekends. Social co-viewing parenting was directly positively associated with children’s TV viewing on weekends, but not weekdays. The strongest influence on children’s TV viewing was having a TV in the child’s bedroom. Negative POETV was weakly associated with having a TV in the child’s room.

**Conclusions:**

These findings suggest that POETV and parenting may have a greater impact on weekend TV viewing, when children tend to watch more TV, than weekday. The models suggest that POETV, parenting and especially removing the TV from children’s rooms may be promising targets for interventions.

## Background

Television viewing is a common childhood behavior [1, 2] that has been linked to a number of positive and negative outcomes. Educational television programs have demonstrated efficacy in teaching children problem solving [3], vocabulary [4], and early reading skills [5]. Other research has demonstrated that television can be an effective medium for promoting health behaviors like smoking cessation [6], eating vegetables [7], and practicing safe sex [8]. However, the prevalence and potentially detrimental effects of television containing violent and/or sexual programming have also been well documented [9–14]. Excessive television viewing during childhood has been linked to conduct problems [15, 16], aggressive behavior [17], and fewer social skills [15]. Children who watch more television and/or have a television in their bedroom are also more likely than other children to experience sleep difficulties [18]; and television viewing generally has an inverse relationship with school performance and academic achievement among children and adolescents [19–21].

Concern about childhood obesity has also prompted some to question the impact of television viewing on children’s food intake and weight status. Research has demonstrated that television use is positively associated with overweight and obesity in children [22, 23], and the relationship may be explained by displacement of physical activity [24], increased caloric intake while viewing [24], and/or exposure to unhealthy food messages/advertisements [25–27]. Several studies have examined programming for children and adolescents and discovered a large volume of advertisements for food items that are high in fat, sugar, and sodium content and/or of little nutritional value [28–30].

Parental restriction is a highly effective means by which to limit television viewing, though it is used inconsistently across households [1, 31–34]. Little is known about what motivates some parents to restrict while others do not. Social Cognitive Theory [35] is often used as a basis for research in health promotion because it outlines the beliefs and attitudes that might serve to motivate and/or facilitate behavior change [36]. Bandura [37] lists outcome expectations as one of the core determinants that aid in translating knowledge to effective health practices, and defines them as the perceived costs and benefits of engaging in a specific behavior. Outcome expectations are thought to serve as either incentives or disincentives for engaging in certain behaviors [36], so it is reasonable to expect that they might influence the parental decision to restrict, or not restrict, children’s television viewing. Understanding how parental outcome expectations for television viewing relate to parenting practices that influence children’s TV viewing would aide in developing effective interventions that ultimately reduce children’s screen time by increasing parental limit setting.

The aim of this study was to examine the relationship among parental outcome expectations measured with a validated measure of both positive and negative TV outcome expectations [38], parenting practices related to children’s television viewing, and children’s actual TV viewing. We hypothesize that parents with more negative outcome expectations concerning television viewing engaged in more restrictive behaviors and have children who watched less television. The opposite is also expected, such that parents who have more positive outcome expectations for television viewing engaged in less restrictive behavior and television viewing played a larger role in family activities (e.g., social co-viewing).

## Methods

### Sample and procedure

Parents of 6–12 year old children were invited to complete an online survey asking in part about their sociodemographic, TV parenting, and child’s TV viewing behavior. Participants were recruited through oral presentations and advertisements posted online and in socio-economically diverse areas of a large metropolitan area. The recruitment procedures have previously been described by O’Connor et al. [38] in more detail. Inclusion criteria required that the participant was the parent or legal guardian of a 6–12 year old child, that he/she lived with the child at least 50 % of the time, that he/she and the child resided in the county where the study took place, and that he/she was able to read and write in English or Spanish. Interested participants accessed the survey online and informed consent was gathered at the beginning with an introductory letter. Participants first responded to a set of screener questions to assess eligibility and, if eligible, had the opportunity to complete questionnaires. Eligible participants who completed the required questions (11 of 12 question sets) were entered into a raffle for one of 15 $100 gift cards. The survey was available on the internet from April 2012 to August 2012.

The survey was accessed 595 times of which 486 agreed to participate. Thirteen data entries were removed that were deemed to be duplicates from 9 participants, and 370 qualified to participate by the screening protocol. Two hundred ninety-nine participants had complete data for this study, but 12 additional participants were removed because the child’s reported age was outside of the inclusion criteria (6–12 years old). Therefore, a total of 287 parents (77.7 % of those who qualified) were included in this analysis (94.1 % female). The diverse sample of children is relatively representative of the study area and consisted of Hispanic (46.0 %), Caucasian (25.4 %), African-American (14.3 %), and other (14.3 %) ethnicities. The parents reported having primarily male children (58.2 %). Demographic descriptors for the sample can be found in Table 1.

**Table 1:** Sample characteristics

Variables	(n = 287)
**Parent/Child characteristics**	
Parent sex, n (%)	
Female	270 (94.08)
Male	17 (5.92)
Parent age, mean years (SD)	37.44 (8.36)
Child sex, n (%)	
Female	120 (41.81)
Male	167 (58.19)
Child age, mean years (SD)	9.29 (2.12)
Household highest education n (%)	
High School Graduate/GED or less	47 (16.38)
Technical School or Some College	87 (30.31)
College Graduate	74 (25.78)
Post Graduate Study	79 (27.53)
Child’s race, n (%)	
Caucasian	73 (25.44)
African-American	41 (14.29)
Hispanic	132 (45.99)
Other	41 (14.29)
TV in child’s room, n (%)	
Yes	156 (54.36)
No	131 (45.64)
**Child TV & DVD viewing in hours/day, mean (SD)**	
Weekday^†^	2.33 (2.43)
Weekend	3.87 (2.31)
**Parent TV & DVD viewing in hours/day, mean (SD)**	
Weekday	2.28 (1.98)
Weekend	3.33 (2.31)
**Parent Outcome Expectations for Child TV viewing** ^$^, summed mean score (SD)	
**Positive outcome expectations**	
Parent-Centered (7 items)	17.06 (5.38)
Child-Centered (5 items)	15.94 (3.85)
**Negative outcome expectations**	
TV and Content Exposure (7 items)	20.03 (6.56)
Prevent Other Activities (6 items)	17.65 (5.59)
**TV Parenting Practices,** ^#^ summed mean score (SD)	
Social Co-viewing (5 items)	18.31 (3.48)
Restrictive (5 items)	18.88 (4.00)

^†^1 participant reporting more than 20 h TV & DVD viewing was removed as (s)he was an outlier with implausible value

^$^response range from 1 (strongly disagree) to 5 (strongly agree)

^#^response range from 1 (Never) to 5 (Always)

### Measures

#### Parent’s outcome expectations

The Parent’s Outcome Expectations for Children’s TV Viewing (POETV) is a 25-item questionnaire consisting of two scales that measure positive and negative outcome expectations related to child television viewing [38]. The scales use the anchor “If I let my child watch TV…” and requires parents to rate various outcomes using a five-point likert-type scale. The positive POETV scale has two sub-factors: Parent-Centered (7 items; e.g., I would have time to do my work) and Child-Centered (5 items; e.g., he/she would learn new things). The negative POETV scale has two sub-factors: TV and Content Exposure (7 items; e.g., he/she would see too much violence) and Prevent Other Activities (6 items; e.g., we would have less time to spend together as a family). Cronbach’s alphas for the subsample included in this analysis indicated that the sub-scales had good internal consistency reliability: Positive POETV Parent Centered α = 0.82; Positive POETV Child Centered α =0.75; Negative POETV TV Exposure and Content α = 0.87; Negative POETV Prevent Other Activities α = 0.83.

#### TV parenting practices

TV parenting practices were measured using the Television Mediation Scale [39], that includes two sub-factors used in this study: Restrictive Mediation (e.g., How often do you set specific viewing hours for your child?), and Social Co-viewing (e.g., How often do you watch a TV program together because you both like a program?). The factors each consist of five items with a five-point likert-type response scale (1 = never, 5 = always). The current study used sum scores from the Restrictive Mediation and Social Coviewing subscales. The Restrictive Mediation (α = .77) and Social Coviewing (α = .88) subscales each demonstrated adequate internal consistency reliability in the current sample.

#### Television viewing

Parent and child television viewing was measured as part of a larger measure of screen media use, based on a modified version of a global weekly TV viewing estimate that had reasonable correspondence to videotaped observations of children’s TV viewing in the home [40]. Participants answered a set of five questions in response to four different prompts: child weekday, child weekend, parent weekday, and parent weekend. The questions assessed: 1) Watching TV on TVs, computers, or other devices; 2) Watching videos or DVD’s; 3) Playing videogames (such as Xbox, Wii, Playstation); 4) Playing hand-held videogames (such as DS, iPod, or Leapster); 5) Using a computer for something other than activities related to school or work (such as browsing the internet, computer games, or e-mail). Parents responded by entering the typical number of hours and minutes per day for each question for themselves and their 6–12 year old child. In the current study, the responses for “watching TV” and “watching videos or DVD’s” were combined to create one “TV viewing” variable. One outlier was removed from the sample due to an implausible number of reported viewing hours (>20 h TV and DVD viewing per day).

#### Television in child’s room

Participants were asked whether or not there is a television in the room where his/her child sleeps at night with a dichotomous response choice.

#### Demographic information

Participants were asked to provide information concerning a number of demographic variables.

### Data analysis

**Fig. 1:**
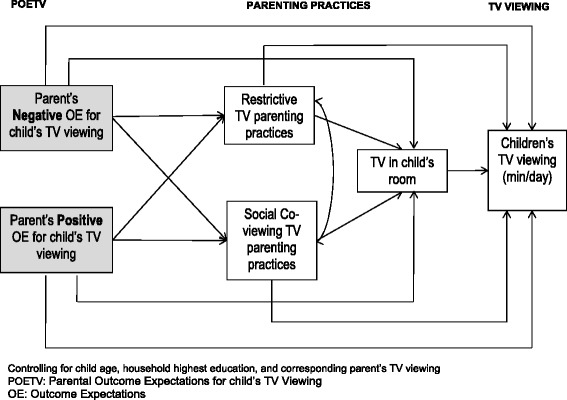
Hypothesized model

The frequencies, percentages, means and standard deviations were calculated for all the demographic variables and study variables. We examined associations of study variables using Spearman and point-biserial correlations. Data were analyzed using path models which is a special case of structural equation modeling. The parent’s positive and negative POETV were hypothesized to have direct and indirect effects on children’s weekday/weekend TV and video viewing (hereafter referred to as TV viewing). The mediators included restrictive TV parenting practices, social co-viewing TV parenting practices, and having a TV in child’s room. Previous research has demonstrated differences in children’s television viewing on weekdays and weekends [41]. To account for potential differences in the variables associated with television viewing, weekdays and weekends were assessed using two separate models. Well-known correlates of children’s television viewing (e.g., age, household education, parental TV viewing) [42–45] were also controlled for. The hypothesized model is depicted in Fig. 1.

In this study, the original hypothesized model was “just-identified”; therefore, degree of freedoms was not available to calculate chi-square goodness-of-fit index. Several steps were followed to conclude the final models. First, the path estimates instead of the model fit indices were obtained by fitting the original hypothesized model to the data. Second, the hypothesized model was revised by removing the non-significant paths one at a time, starting with the least significant. These steps allowed retention of only the significant paths, which resulted in additional degrees of freedom so it was possible to obtain the model goodness-of-fit index. The path models were assessed using Mplus (version 7.4, Los Angeles) with the Mean- and Variance-adjusted Weighted Least Square (WLSMV) estimation. Model fit was assessed by the criteria of Root Mean Square Error of Approximation (RMSEA) ≤ 0.05 [46], Comparative Fit Index (CFI) > 0.95 [47], the Tucker-Lewis Index (TLI) > 0.95, and Weighted Root Mean Square Residual (WRMR) < 1.0 [48].

## Results

### Descriptive statistics

Descriptive statistics are summarized in Table 1. On average, children in this sample viewed more than the recommended two hours of television on both weekdays, 2.33 h (sd 2.43), and weekend, 3.87 h (sd 2.31) days. Parental viewing was similar to that of children’s, with an average of 2.28 (sd 1.98) hours on weekdays and 3.33 (sd 2.31) hours on weekends.

Indirect relationships between variables were examined using Spearman and point-biserial correlations summarized in Table 2. Positive POETV that was Child Centered was positively correlated to social co-viewing (*r*_s_ = 0.134, p < .05) and negatively correlated to restrictive parenting practices (*r*_s_ = −0.138, p < .05). Negative POETV for TV and Content Exposure (*r*_s_ = −0.204, p < .001) as well as Prevent Other Activities (*r*_s_ = −0.281, p < .001) were negatively correlated with social co-viewing. Negative POETV Prevent Other Activities also shared a negative correlation with the presence of a television in the child’s room (*r*_pb_ = −0.298, p < .001). The reader is referred to O’Connor et al. [38] for a detailed description of the correlations among POETV sub-factors as well as between POETV and child TV viewing.

**Table 2:** Spearman and point-biserial correlations

	TV Parenting Practices		Presence of TV in room^a^
	Social co-viewing	Restrictive	
**Positive POETV**			
Parent Centered	0.002	−0.110	−0.008
Child Centered	0.134*	−0.138*	−0.019
**Negative POETV**			
TV & Content Exposure	−0.204***	0.073	−0.096
Prevent Other Activities	−0.281***	0.086	−0.298***

Controlled for child’s age, parent education, and child ethnicity/race

*p<0.05

***p<0.001

^a^Point-biserial Correlation. POETV: Parental Outcome Expectations of child’s TV Viewing

### Evaluation of the weekday model

**Fig. 2:**
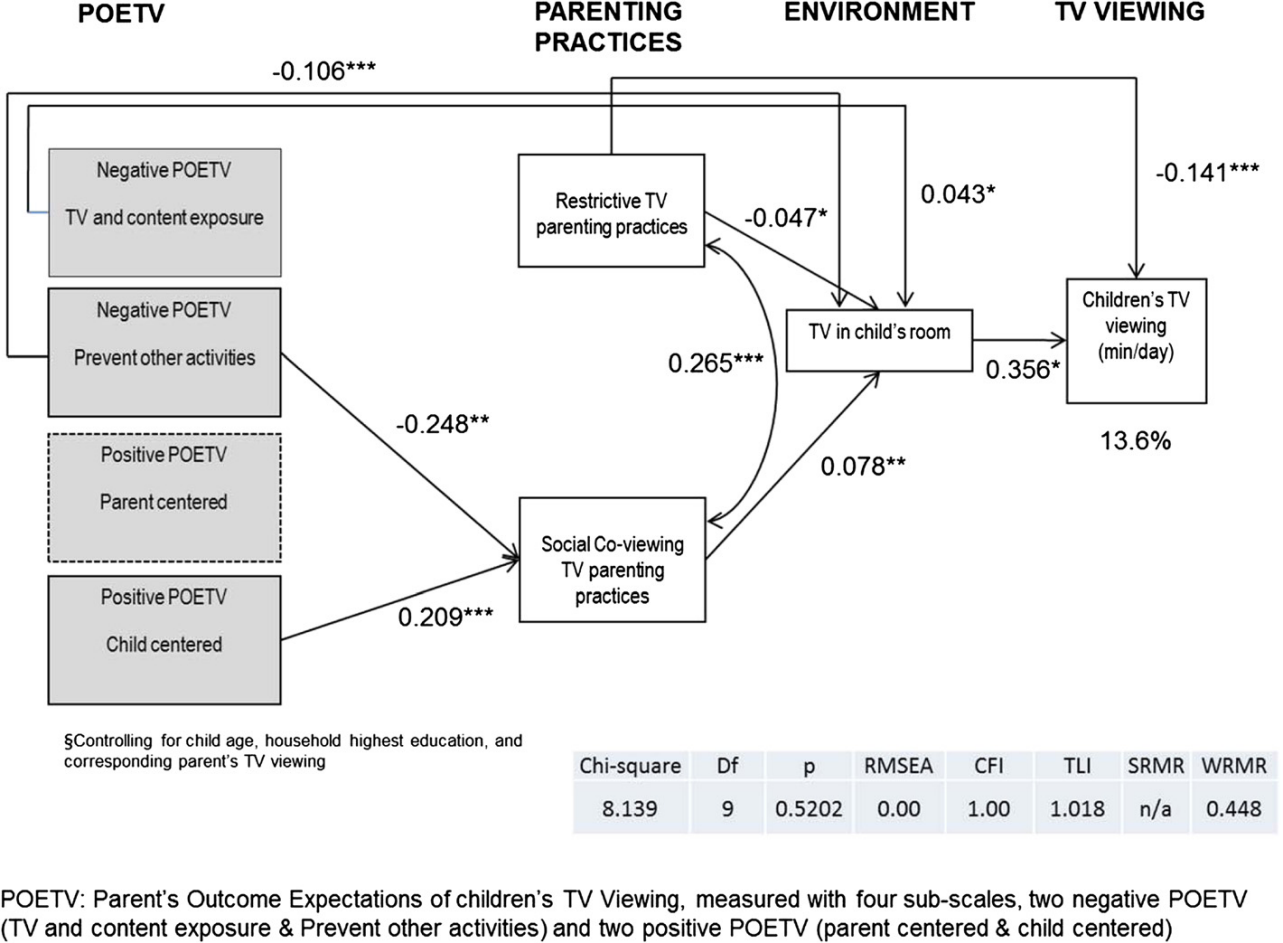
Path model of the association of parent’s outcome expectations and parenting practices on children’s television viewing on “weekdays”

The final model for TV viewing on weekdays is shown in Fig. 2. The model fit was good (*X*^2^ (9, n = 286) = 8.14, p = 0.52, RMSEA = 0.00, CFI = 1.00, TLI = 1.02, WRMR = 0.45), and it accounted for 13.6 % of the variance in children’s television viewing. The model indicates that more negative POETV for Prevent Other Activities was associated with less social co-viewing (−0.248, p < .01), and negatively associated with having a television in the child’s room (−0.106, p < .001). On the other hand, more negative POETV for TV and Content Exposure was weakly positively associated with having a television in their child’s room (0.043, p < 0.05). More positive POETV that was Child Centered was positively associated with greater amounts of social co-viewing parenting (0.209, p < .001). There was a positive association between restrictive parenting practices and social co-viewing (0.265, p < .001). Parents who restrict television more were less likely to have a television in the child’s room (−0.047, p < .05) and had children who watch less TV (−0.141, p < .001). Social co-viewing was positively associated with allowing a television in the child’s room (0.078, p < .01). Finally, the presence of a television in the child’s room was associated with greater amounts of television viewing (0.356, p < .05), and was a mediator for the influence of POETV and parenting practices on children’s television viewing.

### Evaluation of the weekend model

**Fig. 3:**
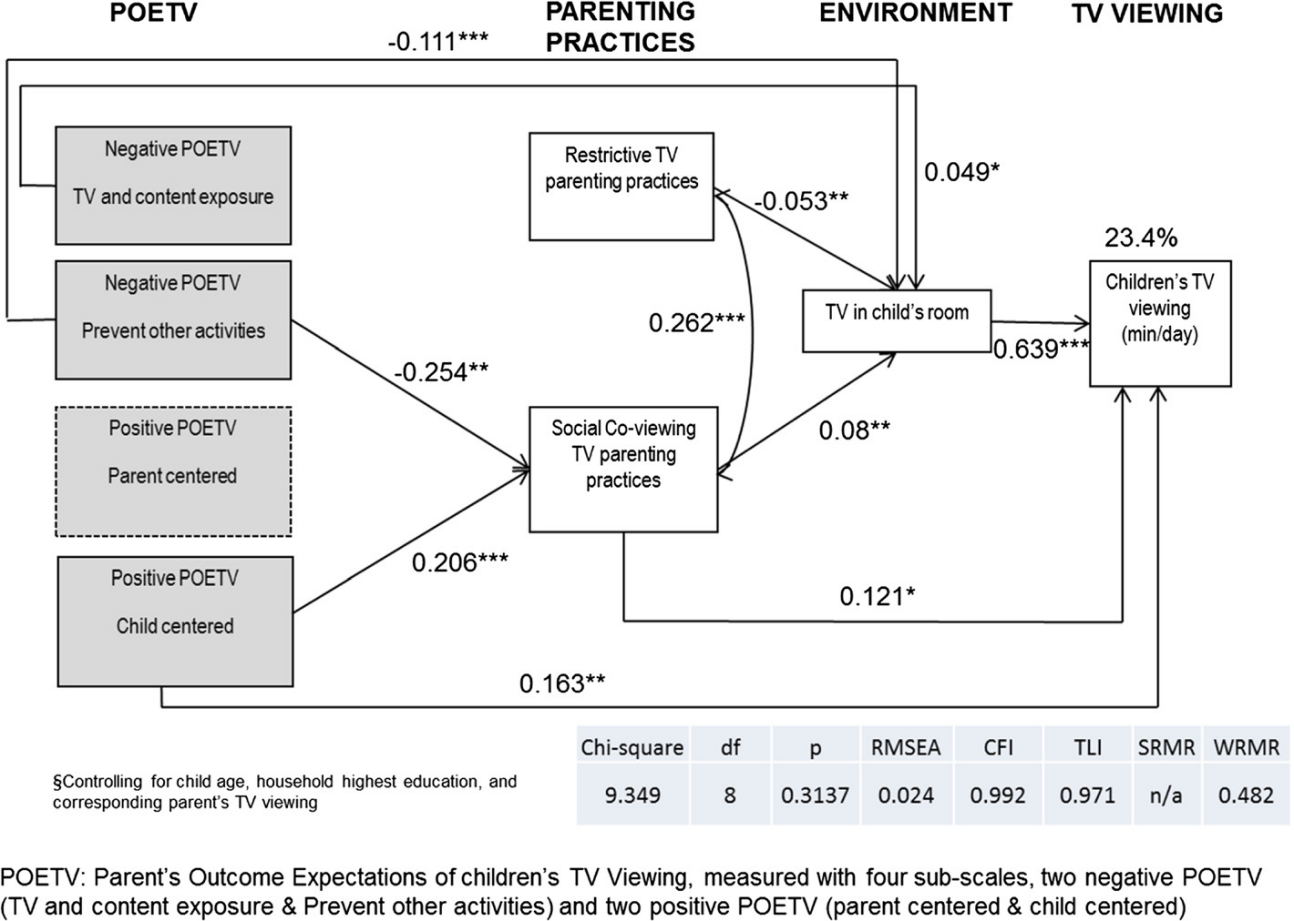
Path model of the association of parent’s outcome expectations and parenting practices on children’s television viewing on “weekends”

The final model for TV viewing on weekends is shown in Fig. 3. The model fit was good (*X*^2^ (8, n = 287) = 9.35, p = 0.314, RMSEA = 0.02, CFI = 0.99, TLI = 0.97, WRMR = 0.48), and it accounted for 23.4 % of the variance in children’s television viewing. Similar to weekdays, the model indicates that more negative POETV for Prevent Other Activities was associated with less social co-viewing (−0.254, p < .01), and negatively associated with having a television in their child’s room (−0.111, p < .001). Again, more negative POETV for TV and Content Exposure was weakly positively associated with having a television in their child’s room (0.049, p < 0.05), and more positive POETV- Child Centered was associated with greater amounts of social co-viewing (0.206, p < .001). Unlike the model for weekdays, in the model for weekends parents’ positive POETV- Child Centered had a direct path to children’s television viewing (0.163, p < .01). However, POETV once again had no association with restrictive TV parenting. There was a positive association between restrictive parenting practices and social co-viewing (0.262, p < .001). Parents who restricted TV were also less likely to have a television in their child’s room (−0.053, p < .01), while those who reported social co-viewing parenting were more likely to have a television in the child’s room (0.080, p < .01). Unlike weekdays, social co-viewing parenting was directly associated with greater child television viewing (0.121., p < 0.05). On weekends, the presence of a television in a child’s room was strongly positively associated with time spent watching television (0.639, p < .001).

## Discussion

The models of weekday and weekend child TV viewing accounted for 14 % and 23 % of the variance in this behavior, respectively. The results provided partial support for the hypotheses, with notable differences between weekday and weekend viewing. As expected, positive Child Centered POETV was directly related to social co-viewing on both weekend and weekdays. Negative Prevent Other Activities POETV was negatively associated with social co-viewing on both weekdays and weekends. Contrary to our hypothesis, the POETV subscales were not associated with restrictive parenting practices on weekdays or weekends. Much of the influence of POETV and parenting practices were mediated through the presence of a television in the child’s room. Only restrictive parenting practices had a direct negative association with children’s television viewing on weekdays, while social co-viewing parenting had a direct positive association with children’s television viewing on weekends. The only direct link of the POETV subscales to children’s television viewing was Positive Child Centered POETV on weekends.

These findings suggest that on weekdays, but not weekends, parental restriction is important in limiting children’s television viewing, though POETV does not directly influence parental restriction. The effectiveness of parental restriction is well documented [32, 33, 49] and additional studies will need to identify what influences parents to restrict their child’s television viewing. On the other hand, social co-viewing parenting and Positive Child Centered POETV appear to be more influential on children’s television viewing on weekends. Previous research has documented that both parents and children tend to watch more TV on weekends than on weekdays [30, 41], a finding replicated in the current study. Weekends tend to contain more unstructured time than weekdays, which may lead children to occupy themselves with activities such as television viewing [50]. Parents who are more likely to view television with their child may have more time to do so on weekends. It is also plausible that parents who typically restrict during the weekdays may be less inclined to do so on weekends because they themselves enjoy watching television. Researchers understand that parental modeling plays an important role in the amount of television children watch [43, 51], so understanding their attitudes, particularly the positive expectations for allowing TV viewing, may help to modify their behavior and, in turn, their children’s.

The effect of parenting practices on both weekdays and weekends was mediated, in part or in whole, by the presence of a television in the child’s room. The relationship between the presence of a television in the bedroom and increased television viewing is well documented in the scientific literature [49, 52, 53]. Qualitative data from previous research [54] have indicated that positive and negative parental outcome expectations are associated with the presence of a television in the child’s room. The current study corroborates that finding with quantitative data, and provides additional information with which to further understand the relationship between POETV and children’s television viewing. The finding that the presence of a TV in the child’s room had the strongest relationship with television viewing on both weekdays and weekends suggests this might be an even more promising target for intervention than parenting practices, such as restriction.

Given that Negative POETV through Prevent Other Activities had a direct negative relationship to the presence of a TV in the child’s room and whether parents used social co-viewing parenting practices in both the weekday and weekend models, suggests that increasing negative POETV may be an effective method for removing TV from child’s room and reducing family co-viewing, both which could impact children’s TV viewing. Positive Child Centered POETV also had a positive association with the use of social co-viewing parenting, so another potential intervention aim may be to decrease Positive Child Centered POETV. An intervention which actually targeted parental outcome expectations in an effort to reduce child television viewing among preschool children found that increasing negative outcome expectations for TV viewing was associated with significantly reduced child TV viewing over the course of the study [55]. The intervention described by Zimmerman et al. [55] included monthly newsletters containing information about behavior change and child television viewing, as well as monthly contact with a case manager who reminded caretakers about the potentially detrimental effects of excessive TV viewing and worked with them to develop strategies for modifying their children’s viewing behaviors [55]. Future studies would benefit from evaluating the impact that providing information on the potential negative impacts of child TV viewing has on parent’s positive outcome expectations, along with the benefit of restricting children’s TV viewing on parent’s negative outcome expectations.

The current study makes several important contributions to the literature on child television viewing. Considering the role of parental outcome expectations in child television viewing is a relatively new concept and the subject of few empirical investigations to date. Furthermore, this investigation used a validated measure of parental outcome expectations rather than the “convenience scales” previously employed to evaluate this construct [55]. Finally, the use of path analysis, a type of structural equation modeling, as a means of analysis enabled the researchers to simultaneously evaluate the relationships among multiple variables assessed via the hypothesized models. This allowed for investigation not only of the relationship between POETV and parenting practices, but also POETV and other constructs known to be related to children’s television viewing (presence of TV in child’s room).

However, the study is not without limitations. A subjective, recall-based measure of TV viewing was used, which could lead to inaccurate reports due to faulty recall and/or social desirability bias. Content of what the child viewed was not assessed which may impact parent’s outcome expectations of allowing their child to watch TV. Future research would benefit from including a more objective measure of television use (e.g., electronic monitoring), and assessing the programs and content viewed by children on TV. Additionally, participation in the study required computer literacy and Internet access, which limits the generalizability of these findings to such families. Fathers were underrepresented in the current sample and the county from which the data were collected is not nationally representative. All of these factors limit the generalizability of these results.

## Conclusions

The current study offers a framework with which to conceptualize and execute additional investigations of how parental outcome expectations might influence children’s television viewing. This research suggests that positive and negative POETV may be important parental attitudes to address in interventions focused on reducing children’s television viewing, though additional research is necessary to understand the most effective methods for doing so. Removing the television from the child’s room is an important intervention aim, and the findings suggest that increasing Negative POETV may aid in accomplishing this goal. The weekend and weekday differences in parental television practices and children’s television viewing observed in the current study suggest that interventions may need to target weekday and weekend television viewing differently.

## Abbreviations

TV: Television
POETV: Parent’s outcome expectations for children’s TV viewing
WLSMV: Weighted least square mean variance
RMSEA: Root mean square error of approximation
CFI: Comparative fit index
TLI: Tucker-lewis index
WRMR: Weighted root mean square residual
